# PyCCAPT: A Python Package for Open‐Source Atom Probe Instrument Control and Data Calibration

**DOI:** 10.1002/jemt.70011

**Published:** 2025-07-19

**Authors:** Mehrpad Monajem, Benedict Ott, Jonas Heimerl, Stefan Meier, Peter Hommelhoff, Peter Felfer

**Affiliations:** ^1^ Institute for General Materials Properties, Department of Materials Science & Engineering Friedrich‐Alexander Universität Erlangen‐Nürnberg (FAU) Erlangen Germany; ^2^ Department of Physics Friedrich‐Alexander‐Universität Erlangen‐Nürnberg (FAU) Erlangen Germany

**Keywords:** atom probe control, atom probe data acquisition, atom probe raw data analysis, atom probe tomography, data calibration

## Abstract

Currently, the vast majority of atom probe instruments in use are commercial systems with closed, proprietary software. This is limiting for many experiments where low‐level access to machine control, experiment data, or custom instrument setups is necessary. Over the past decade, advancements in off‐the‐shelf detector systems, fast data bus systems, and the availability of high‐level programming languages such as Python have made it feasible to design and construct atom probe systems without extensive engineering expertise. Despite this progress, developing control system software, associated instruments, and data calibration algorithms remains a significant challenge for many projects. In this article, we introduce an atom probe control system that can be flexibly adapted to various hardware configurations. This system also includes essential instrument and experiment calibration algorithms, offering complete transparency to the user. This framework provides flexibility for innovative experiments and enhances calibration accuracy not possible with commercial systems. The methods and algorithms discussed are implemented in Python Control and Calibration for Atom Probe Tomography (PyCCAPT), which is an open‐source solution for APT, addressing a gap in experimental control and data processing. While not compatible with commercial atom probes for data acquisition, its calibration module can be used for direct‐flight‐path systems and adapted for reflection‐based instruments.

## Graphical abstract

Open‐source PyCCAPT software enables flexible atom probe control and calibration, offering transparency and adaptability for diverse hardware setups. By integrating advanced algorithms, it enhances experiment precision and supports innovative research beyond the limitations of proprietary systems. Open‐source PyCCAPT software enables flexible atom probe control and calibration, offering transparency and adaptability for diverse hardware setups. By integrating advanced algorithms, it enhances experiment precision and supports innovative research beyond the limitations of proprietary systems.

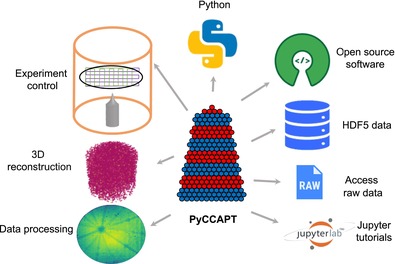

**Summary**

- In this work, we introduce PyCCAPT, a Python‐based, open‐source package designed to address the limitations of proprietary atom probe tomography (APT) systems.
- PyCCAPT provides complete access to instrument control and data calibration, offering researchers flexibility and customization for APT experiments.
- By combining modern software practices with accessible hardware configurations, PyCCAPT enables innovative experimental setups and improved calibration accuracy that were previously unattainable with commercial systems.
- PyCCAPT functions as an integrated control and data acquisition platform, supporting real‐time visualization, post‐processing steps such as 3D reconstruction, and advanced calibration algorithms.
- Its modular architecture supports seamless integration with a wide range of custom hardware setups, allowing researchers to adapt the system to their specific experimental needs.
- Moreover, PyCCAPT offers real‐time control over key experimental parameters, such as voltage and laser settings, making it suitable for any custom experiment control.
- This transparency and adaptability empower researchers to overcome the constraints of proprietary tools, enabling rapid adoption of novel experimental designs while maintaining full control over data acquisition, calibration, and analysis workflows.
- By bridging the gap between commercial systems and the need for customizability in APT research, PyCCAPT lays the foundation for future advancements in both instrumentation and data analysis.
- Its open‐source nature fosters collaboration, innovation, and reproducibility, making it a valuable resource for the APT community.

## 1 Introduction

**FIGURE 1:**
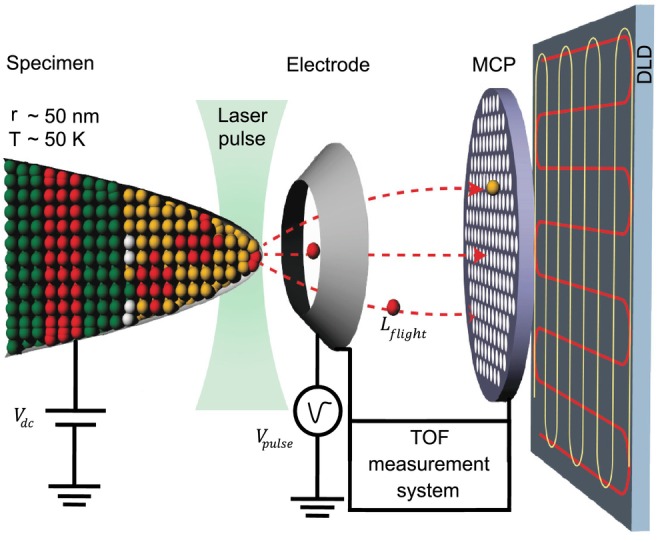
Diagram illustrating the APT instrumentation; the diagram depicts the APT instrumentation situated within the ultrahigh vacuum analysis chamber. This representation, while not to scale, offers a depiction of the straight flight‐path configuration.

Atom probe tomography (APT) is a unique material characterization technique capable of delivering three‐dimensional (3D), atomic‐level analysis with nanometer resolution (Gault et al. 2009) on the basis of time‐of‐flight (TOF) mass spectrometry (Barofsky and Müller 1968; Blavette et al. 1993). APT allows for imaging and unbiased analysis of all chemical elements in a sample with equal sensitivity. This technique has been applied to a variety of materials, from high‐strength structural alloys (Le Breton et al. 2013) and semiconductor materials (Lauhon et al. 2009), to catalyst nanoparticles (Barroo et al. 2020) and even to metal–organic frameworks (Ji et al. 2020). To perform APT, sharp needle‐shaped specimens are placed in an ultrahigh vacuum environment at cryogenic temperatures under a high electric voltage. The sample is then exposed to an intense voltage pulse (VP) (typ. < 5 ns) (Barofsky and Müller 1968), or a laser pulse (LP) (typ. < 10 ps) (Gault et al. 2006; Kellogg 1981). Pulsing causes individual atoms or molecules to be field evaporated as ions from the tip surface at a very defined time, which in turn can be correlated with their arrival time at a 2D time‐resolved detector, allowing TOF measurements and tomographic reconstructions (Bas et al. 1995). APT can be used to identify both light and heavy elements, with no fundamental restriction, as long as pulsing intervals allow for a sufficient flight time for the generated heavy ions. Figure 1 shows a schematic of the atom probe instrumentation and the evaporation process. Although 1D atom probe (Müller et al. 1968) and APT (Blavette et al. 1982) have been around for decades, the most dramatic improvements have been made in the 2000s, with the introduction of wide‐angle detector (Deconihout et al. 2007), reflectron (Sijbrandij et al. 1996), and laser‐pulsed APT (Gault et al. 2006; Blavette et al. 2008; Schlesiger et al. 2010), as well as specimen preparation based on focused ion beams (Larson et al. 1998). This has drastically increased ion collection rates from several hundred ions per second to > 10^4^. At the time, this rate pushed the available data processing hardware to its limits.

In recent years, the scope of APT applications has expanded significantly, leading to its widespread adoption in various research fields. Achieving high data collection rates in APT has necessitated specialized knowledge in custom electronic design and the simultaneous control and readout of multiple hardware devices, which can be challenging to implement. Over the past two decades, the majority of APT research has been conducted using commercial APT systems, with the local electrode atom probe (LEAP) series from CAMECA (CAMECA Instruments Inc., Madison, USA) dominating the market. In fact, these instruments are the result of the merger of three commercial atom probe companies that existed in the previous decade: Oxford Nanoscience (Kindbrisk) (Cerezo et al. 1988) (Oxford, UK), CAMECA France (Blavette et al. 1993) (Gennevilliers, France), and Imago (Kelly and Larson 2000) (Madison, WI, USA). It should be noted that Inspico (Technologie‐Transfer‐Initiative GmbH, Stuttgart, Germany) has also developed a commercial atom probe, although its user base is smaller compared to CAMECA's, and it also operates with a closed codebase.

The access to easy‐to‐operate commercial instruments with a very wide scope of application was one of the main reasons for the rapid adoption of this technique in the past decade. However, there are still cases where the nature of the experiment requires a custom setup or direct access to the raw data (Ismail et al. 2005), even though research‐dedicated instruments are generally expected to provide such access. A custom setup may be a unique vacuum system (Felfer et al. 2022), a new laser technology (Chiaramonti et al. 2019), a setup for an in situ experiment (Maillet et al. 2024) or a custom experiment control algorithm. Recently, the improvements in electronics and computational speed have made it possible to build a system running on easy‐to‐use consumer‐grade hardware (e.g., USB 3.0) and scripted programming languages such as Python. This makes it possible for individual researchers to build a system from off‐the‐shelf parts, without the need for specialized electronics knowledge, giving full access to all aspects of the experiment. Despite these advancements, programming the software infrastructure to drive the experiment remains a time‐consuming challenge. In this paper, therefore, we introduce an open‐source Python package, PyCCAPT,^1^ that provides the software infrastructure for such a system, from data acquisition to data calibration and reconstruction. This gives the ability to avoid the lengthy software development process and concentrate on the setup of the experiment. It gives full access to all algorithms controlling the experiment, as well as full‐depth access to the experimental data.

While commercial APT systems have made significant strides in accessibility, they remain restrictive for those requiring low‐level control, transparency, or integration of novel experimental setups. PyCCAPT provides a complete control suite and data calibration functionality in Python which interfaces with hardware DLLs of the detector, vacuum system control, and high‐voltage supplies, where necessary. With this package, any interested researcher can control an APT system, based on both currently commercially available delay line detector systems RoentDek (RoentDek Handels GmbH, Kelkheim‐Ruppertshain, Germany) and Surface Concept (Surface Concept GmbH, Mainz, Germany), with both voltage and laser pulsing. PyCCAPT cannot be used for data acquisition from commercial systems due to their closed hardware architecture and lack of access to time‐to‐digital converter (TDC) data, but it is capable of data processing and calibration. The PyCCAPT package covers experiment control, instrument calibration, mass spectrum calibration, and 3D reconstruction. In this paper, we outline the architecture of PyCCAPT, the implementation, and use cases where a custom setup or low‐level access enables research that is not easily possible with commercial closed‐source instrumentation.

## 2 Materials and Methods

### 2.1 Experimental Setups

We employed two different instruments: a VP and an LP atom probe, both of which were controlled using the Python package presented here. The VP atom probe was developed to achieve low hydrogen vacuum levels (Felfer et al. 2022). Meanwhile, the LP atom probe is at the Laser Physics Department at FAU and features an ultrashort LP. The VP atom probe, detailed in prior research, features a titanium analysis chamber following a typical three‐chamber structure that includes a load lock, a buffer chamber, and the analysis chamber. This atom probe prioritized achieving a near‐H‐free vacuum environment, with particular emphasis on optimizing the vacuum system within both the analysis chamber and the sample stage.

**FIGURE 2:**
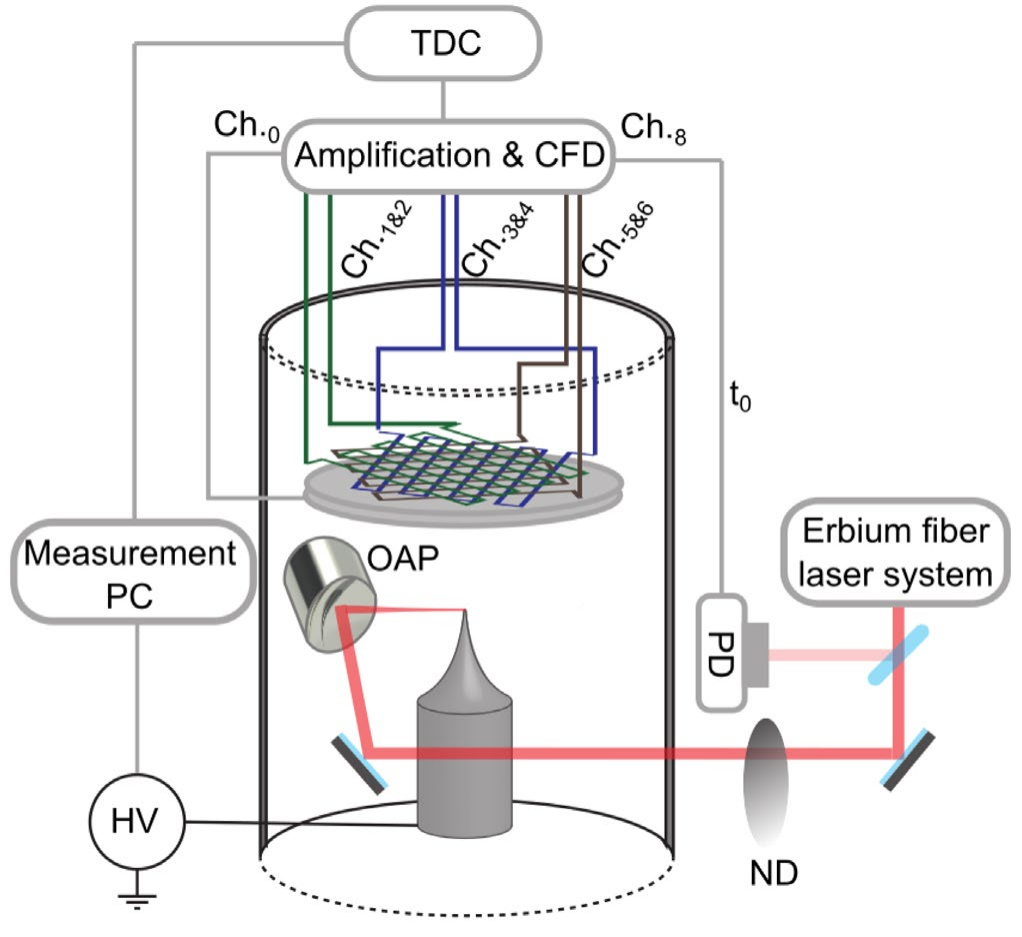
LP atom probe system; the laser systems and electronics shown are the APT system with fs laser triggering. The components are explained in the main text.

The LP atom probe chamber was originally designed to study the emission process and dynamics of electrons triggered by fs LPs. The typical electron energies investigated in this chamber are around a few tens of eV (for more details on the electron emission experiments, see Meier et al. 2023). Therefore, almost no electron optics is present. Only the specimen is at a given potential without using a counter electrode. The detector entry is at ground potential, together with the chamber wall, and all detector fields are shielded. The detector, in this case, is a RoentDek hexanode DLD, which is connected to the measurement PC via a PCIe connection. The structure of the chamber can be seen in Figure 2, which consists of a small load lock and an analysis chamber. For APT experiments, we implemented the ability to supply positive high voltage up to 20 kV. The used light source is an Erbium fiber (Toptica GmbH, Tokyo, Japan) laser with a central wavelength of 1550 nm, a pulse duration down to 12 fs, and a repetition rate of 80 MHz. This repetition rate is reduced by a Pockels cell down to 100 kHz. The LPs are focused in the vacuum chamber by an off‐axis parabolic mirror (OAP) onto the specimen. The OAP is situated on a three‐axis closed‐loop piezo stage (SmarAct, Oldenburg, Germany). In focus, the beam waist (1/*e* ^2^ intensity radius) is around 4–5 μm. This focus allows us to achieve intensities up to 6 × 10^12^ W/cm^2^. Using motorized neutral density wheels (ND) the pulse energy can be attenuated. This setup creates a rather basic laser APT system.

### 2.2 Data Acquisition

The basic requirements for an APT control system are the control of high voltage, pulse amplitude, and laser parameters during an experiment, as well as the recording of their current values and the collection of the detector data. In addition to this, the recording of vacuum parameters, control of the vacuum level, and cooling system are desired, albeit not necessary. For microelectrode type instruments, control software should also include specimen alignment with respect to the aperture of the microelectrode. From an experimental point of view, the control of the high voltage and depending on the experiment type pulse voltage evolution and laser parameters constitute the core of the control system. By giving the user full access to the control algorithms, they can be optimized for the experiment at hand, and new types of experiments can be incorporated. The dynamic optimization of control algorithms and parameters has to an extent already been incorporated into existing software, for example, by providing different sets of control algorithms for the very early stages of the experiment, the non‐steady‐state initial phase, and the steady state of constant ion flux. This is implemented in the control system of the CAMECA atom probe systems. Other commercial systems such as Inspico atom probes do not make this distinction to this extent.

The acquisition control algorithm is designed to regulate the high voltage and pulse amplitude or laser power of the specimen in order to maintain a consistent number of atoms detected within each acquisition time window. In our case, this is achieved through either a dual proportional algorithm with different constants for voltage increases and decreases or a PID controller to determine the necessary change in the high voltage of the specimen. Therefore, the control can quickly decrease the voltage if too many ions are detected, in order to reduce specimen fractures and ion losses in the detection system, but slowly increase the voltage to avoid overshoots.

To achieve this objective, our control loop uses different proportionality values for voltage increase ($S_{f_{\mathrm{up}}}$) and voltage decrease ($S_{f_{\text{down}}}$). The TDC is used to read the number of hits recorded since the previous TDC reading, via either USB 3.0 in Surface Concept TDC or PCIe in RoentDek TDC. Based on the number of hits registered during the last acquisition time window of the TDC, the high voltage and pulse amplitude are adjusted accordingly. Note that the specifics of the algorithm may vary depending on the experimental requirements and the system used. The upward or downward DC voltage step, $V_{s}$, of high voltage is calculated as follows:

(1) $$V_{s}=S_{f_{\mathrm{up}/\text{down}}}\times \left({\mathrm{DR}}_{t}-{\mathrm{DR}}_{c}\right),$$

where the current detection rate, DR_c_, is

(2) $${\mathrm{DR}}_{c}=\frac{\sum \#\text{Event}}{f}$$

and DR_t_ represents the target detection rate specified by the user and *f* is the pulse repetition rate. DR_c_ is averaged over one control loop period. The set detection rate is not reached instantaneously. The speed at which the set point is reached, as well as the tendency to overshoot or undershoot the target, are typical issues in control algorithms (Li et al. 2006).

The control loop must be executed with sufficient frequency (typ. > 1 Hz) to enable acceptable control dynamics and be responsive to user input. The detector and other electronic devices' maximum response time can also be a limiting factor for the iteration frequency. Historically, the control loop was often done using real‐time control systems with low time jitter, but the performance of modern high‐level programming languages on current PC hardware allows for sufficiently low time jitter to operate a stable control loop, with the frequency being adjustable. Especially the increase of the USB polling rate to 1 kHz has virtually eliminated time jitter issues at the typical control loop frequencies in APT. In our system, control loop frequency is variable, but we found 10 Hz to be sufficient in our experiments. Frequencies of up to 50 Hz were possible before running into quantization artifacts due to a low number of collected ions in each loop iteration. Nevertheless, the control loop frequency can be adjusted and increased as needed based on the specific instrument parameters.

In PyCCAPT, the control algorithm is easily accessible and can be changed at any time to fit a specific experiment. The experimental parameters can also be scripted for a series of experiments in the control software, enabling the user to carry out experiments with varying sets of parameter settings on a specimen, with each set being terminated by one or more end conditions.

### 2.3 Data Processing and Reconstruction

The fundamental principle of chemical identification in APT is TOF mass spectrometry. From the TOF, a mass‐to‐charge (mc) value can be calculated (Kelly and Miller 2007). However, even for identical ions, the TOF varies due to the evolution of the specimen bias voltage as well as the electrostatics and geometry of the instrument. While these variations can easily be calculated for an idealized instrument, or simulated for a known instrument geometry (Vurpillot et al. 2018), in reality usually they are determined based on experiments. This is due to the many variables such as detector entry potential, specimen voltage, and complex geometry boundary conditions. In an idealized instrument (field free drift region), for the influence of the bias voltage, the kinetic energy of the ion with mass *m* and charge state *n* after being accelerated to potential difference *V* of the specimen to the field‐free region is taken as equal to the kinetic energy of the ion. This ion then travels for a defined distance *L*. This leads to the expression

(3) $$\frac{m}{n}=2\mathrm{eV}{\left(\frac{t_{\mathrm{obs}}-t_{0}}{L_{\text{flight}}}\right)}^{2},$$

where $t_{\mathrm{obs}}$ is the observed TOF, $t_{0}$ is the time shift, *V* is the specimen voltage, $L_{\text{flight}}$ is the flight distance, and e is the elementary charge of the electron (Miller and Smith 1987). The mc ratio is usually expressed in Daltons (Da). For measuring mass resolution, the mass resolving power (MRP), defined as m/dm, of an atom probe is a crucial metric in evaluating the resolution of the TOF or mc histogram as a spectrometer for mass or time. However, achieving a high MRP involves several complexities that can affect the accuracy and reporting of its value (Oltman et al. 2011). The mass resolution is often calculated full width at half maximum (FWHM), but can also be calculated at 1/10th, 1/100th maximum, or at any height. We refer to it at half maximum as MRP(0.5).

Even for the simplest and most commonly used atom probe setup, which includes a specimen and a stage at a bias voltage, a counter electrode with the pulse voltage applied, and a detector, there are significant deviations from the idealized view. First, the specimen bias with respect to the counter electrode varies with time when the pulse is applied, leading to variations in ion energy depending on the exact time of departure of the ion (Rousseau et al. 2020). In addition, the bias potential for the microchannel plate (MCP) amplifiers in the detector is typically applied such that the back potential—that is, the potential at the anode—is grounded, while the entrance potential is set to around 2–3 kV for a two‐MCP stack. This simplifies the electrical design of the detector and attracts ions to the detector, effectively enlarging the field of view. However, this is not always the case as, for example, the LP atom probe in this study has a ground potential at the front, that is, an actually field‐free drift region.

After each experiment, the raw data from the detector and the other sensors of the instrument need to be consolidated into a physically meaningful form. This demands some global calibrations, specific to the instrument setup and some per‐experiment calibrations. The global instrument calibrations put the data in the context of the current setup. This includes the calculation of the ion flight times from the raw TDC timing data including propagation delays, and the calculation of the ion impact location from the unscaled TDC output. The former is simply a constant factor, while the latter requires some calibration. Once the physical impact on flight time data is calculated, the experiment‐specific calculations need to be made to create mc ratios from flight times and 3D reconstruction from 2D detector coordinates and arrival sequences. These “corrections” are often separated into two main contributions, the specimen bias voltage (“voltage correction”) and the ion launch angle (“bowl correction,” due to the flat shape of the detector). To this end, iterative fitting procedures are used, since the combination of the specimen geometry varying between and during experiments and the non‐trivial electrostatic environment, has so far made simulation‐based approaches less practical.

The first step in data calibration for APT involves determining the time offset, *t*_0_, which accounts for propagation delays in electronic processing and the actual arrival of the voltage or LP on the specimen. This can be calculated from the data, particularly when using mono‐isotopic or nearly mono‐isotopic elements like Al or Co. By rearranging Equation (3), *t*_0_ and the flight path distance can be determined, provided peak identities are known (Sebastian et al. 2001). Accurate calibration requires identifying well‐spaced peaks in the mass spectrum, with multiple mc values present. To improve TOF separation and minimize angular flight path variations, calibration should focus on small detector sections and narrow voltage ranges.

**FIGURE 3:**
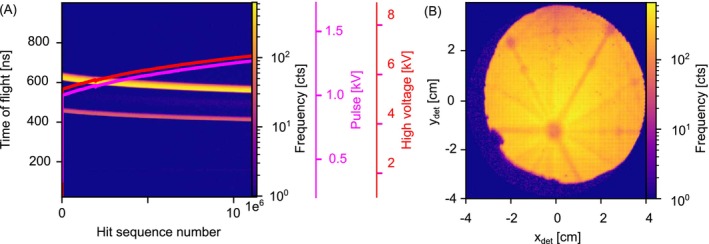
ROI selection; visualization of data cropping and ROI selection in atom probe experiments. (A) A 2D histogram shows the TOF versus ion sequence number alongside high‐voltage and pulse voltage curves, illustrating the evolution of the experiment. (B) The FDM highlights the spatial distribution of ions on the detector. The ROI is defined by selecting a specific ion sequence range and corresponding detector region to exclude artifacts, optimize calibration accuracy, and ensure reliable data reconstruction.

Accurate calibration and data analysis also require limiting the analytical volume to relevant data areas, particularly in microelectrode atom probes. This exclusion prevents complications from the rim of the counter‐electrode and localized field emission, which can negatively affect calibration, reconstruction, and processing algorithms. While including all detected data may enhance compositional variation measurements, accurate calibration often takes precedence. Artifacts should be excluded by considering factors like the voltage curve shape, experiment evolution, background changes, and analysis conditions. Figure 3 illustrates this process, showing a 2D histogram of TOF versus ion sequence number and a field desorption map (FDM) of an Al specimen. The region of interest (ROI) is selected based on the ion sequence range and the detector's ROI.

Due to the geometry of APT instruments and the need to raise the specimen voltage during the experiment, the observed TOF, *t*_obs_, depends on the ionization voltage and the location of the impact on the detector. In addition, because of the detector and the point source of the detected ions, the flight distances—and consequently the TOF for a straight flight path instrument—increase outward from the detector's center, although in other configurations, different trajectories may dominate (Heller et al. 2024). This can be accounted for either before converting the TOF to a mc value or after. Although in current commercial instrumentation by CAMECA, this is done after conversion; we created a reference state, where all TOF values are in reference to a fixed detector position at a fixed voltage. Both approaches are equivalent.

To achieve this aim, a polynomial approximation is commonly used to fit the dependencies of the detector location (bowl correction) and the voltage level of the specimen (Larson et al. 2013). A purely physical approach is somewhat difficult, as the drift region is usually not field‐free. Typically, the detector entry plane is at an elevated potential of approximately 500 V. In systems where the detector entry potential is ground, that is, the drift region is field‐free, the deviation from the ideal angular and voltage corrections is minor. For all other instruments, at least in the straight flight path configuration, the resulting curvature of the flight path results in the necessity of a fitting of the angular and voltage variations. Nevertheless, a simple physics‐based model of a field‐free drift region can be used as a starting point for further optimization. As a result, to eliminate the variation caused by ionization voltages and hit locations, the calibrated TOF, *t*_c_ can be formulated as follows:

(4) $$t_{c}=\frac{t_{\mathrm{obs}}-t_{0}}{C_{V}\left(V\right)C_{B}\left(x_{\det},y_{\det}\right)},$$

where the function $C_{V}\left(V\right)$ takes into account the voltage‐dependent variations in TOF, which can be influenced by various factors such as oversimplified electrostatic assumptions, linear detector responses, and detector bias (Larson et al. 2013). On the other hand, $C_{B}\left(X,Y\right)$ considers differences in the flight path length based on the detector hit coordinates.

In order to carry out the optimization, a region in the TOF spectrum is used, in which the vast majority of detector events can be ascribed to the same ionic species, that is, “ideal” TOF. This is used to maximize MRP iteratively. An appropriate peak should ideally include ions distributed across the entire field of view to ensure a successful optimization algorithm for the flight path calibration (bowl calibration). The parameters defining the voltage correction $C_{V}\left(V\right)$ are then optimized, followed by the parameters defining the bowl correction $C_{B}\left(x,y\right)$. These voltage and bowl optimizations are iterated until MRP stops improving by a predefined threshold. Once the voltage and bowl corrections have been satisfactorily completed, the conversion from TOF to mc is performed using a second‐order parameterized function.

**FIGURE 4:**
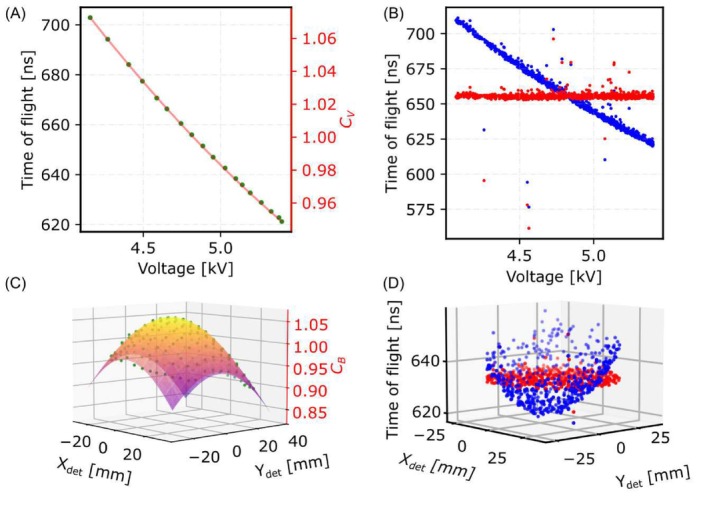
Voltage and bowl correction: (A) The second‐order line is fitted to each data point, with each green point representing a distinct window. These windows can be defined based on either ion sequence or voltage range. The TOF value for each data point corresponds to the peak TOF within its respective window divided by the peak TOF for the selected peak, while the voltage value is computed as the mean of data points within the peak position plus and minus one bin. (B) Blue points show the uncalibrated TOF of the selected peak, which decreases with increasing voltage. Red points show the TOF after calibration using the correction factor $C_{V}$. (C) is fitted to partitioned data points of the selected peak. Each green dot represents the peak TOF of ions within one partition, normalized by the peak TOF of the selected peak. (D) Blue points depict TOF data before bowl calibration, exhibiting a bowl‐shaped pattern due to flight path variations. Red points show TOF after correction, reducing variation based on the detector hit position.

For fine correction, we introduced a fitting method for mc calibration. For voltage calibration rather than solely fitting a polynomial function to all ions within the selected peak, we adopted a sampling approach based on either hit sequence or voltage. This involved segmenting our data into intervals, for example, 1000 ranges based on the hit sequence or voltage. Within each range, we determine the most frequently occurring TOF or mc value by constructing a histogram and pinpointing the maximum peak. Normalizing this peak maximum by the mean of the entire ions that are under the current range plus and minus one bin. We repeated this process for all ranges. This strategy effectively reduces the number of data points, mitigates the impact of noise, and helps to prevent overfitting. Figure 4A shows the line fitted to the Al^+^ TOF ranges, as well as the results after applying the correction factor to the TOF peak (Figure 4B).

For bowl calibration, we employed the same approach by partitioning the ion data based on the detector hit location. This involved creating a mesh on the detector surface with fixed dimensions, that is, 9 × 9 mm. Within each rectangle of this mesh, we identify the highest frequency of occurrence among the partitioned data. By normalizing these values by the mean of all ions within the selected peaks, we then fitted a bowl‐shaped function to these data points. Figure 4C showcases the bowl fitted to the sampled data points of the main peak from an Al sample, while Figure 4D depicts the resulting TOF after bowl correction.

Obtaining a 3D reconstruction of ionized atoms is an essential step in APT analysis. To achieve this, several methods have been implemented, including the method proposed by Bas et al. (1995) and those published by Gault et al. (2011). Similar to commercial software, the retrieved data obtained with PyCCAPT can be used to reconstruct the 3D structure, which is not the focus of the work here.

### 2.4 Software Architecture

**FIGURE 5:**
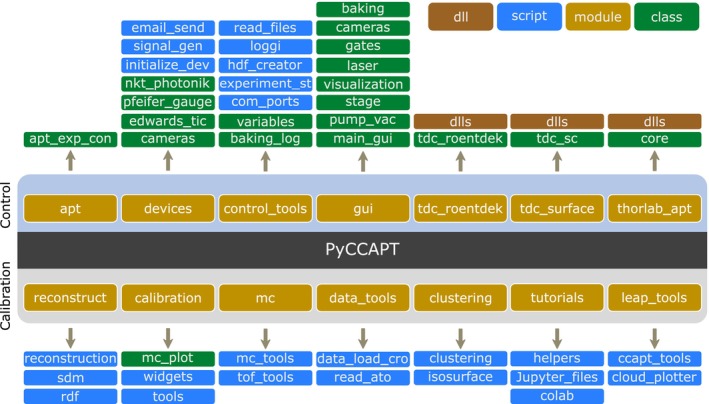
PyCCAPT modules structure; the package modules are detailed in the main text.

The PyCCAPT package is designed with modularity and flexibility, making it straightforward to integrate new devices into the control system. The package consists of two main modules: “control” and “calibration,” as illustrated in Figure 5.

The “control” module contains several sub‐modules that facilitate the control of the APT instrument. The “apt” sub‐module manages experiment control, employing multiprocessing for detector data acquisition and multithreading for updating devices like the high‐voltage power supply and pulser. This parallel execution enables refresh frequencies of up to 50 Hz for the experiment control loop in our instruments, although this limit may vary depending on the response times of individual atom probe devices. This module saves experimental data, including detector raw data, experiment metadata (such as analysis temperature), and supplementary information such as camera images and the status of the graphical user interface (GUI). While these supplementary details are not directly saved in the hierarchical data format (HDF5) file, they are stored separately for easy access. The control module features a main GUI that allows users to control the instrument and manage experiments.

The “calibration” module is focused on data processing functionalities and contains several Jupyter notebook workflows (Jupyter 2023), tailored for tasks such as instrument calibration, data calibration, ranging, and 3D reconstruction. Although it is possible to use the calibration module directly in a Python script, implementing these workflows in Jupyter Notebooks provides interactive exploration and ease of use for users. The Jupyter notebooks also enhance collaboration and scalability by allowing users to work on the notebooks remotely. Furthermore, they can be easily shared across platforms, facilitating collaborative development and review. The calibration module outputs a data structure similar to commonly used APT data formats, such as EPOS or POS, with the addition of extra information like raw data. PyCCAPT also offers the ability to convert its native data structure to EPOS and POS formats. All workflows can utilize common APT data formats such as APT, ATO, EPOS, POS, and CSV, which can be loaded for further analysis or visualization. Detailed information on the data structure is available in the Supporting Information and in the project repository.

### 2.5 Materials

APT data presented in this work were collected from pure Al (99.9%; Goodfellow Cambridge Limited, Huntingdon, UK), ferritic stainless steel X14CrMoS17 (SS‐1.4104) and austenitic stainless steel X2CrNiMo17‐12‐2 (SS‐1.4404) (Zivipf Inc., Treuchtlingen, Germany). These specimens were prepared by electropolishing in 25% perchloric acid in acetic acid, followed by fine polishing in 4% perchloric acid in butanol. The LP atom probe experiment was carried out with pure W (99.9%, Goodfellow), electropolished with 5% KOH in deionized water.

## 3 Results and Discussion

In order to evaluate the effectiveness of our experiment control and data processing algorithms, we conducted measurements on Al, as well as ferritic and austenitic steels using the VP atom probe, as well as W using the LP atom probe. Furthermore, we compared the calibration results of PyCCAPT and the AP Suite a W and 6xxx series Al‐alloy dataset from the LEAP 5000 XS instrument. The results of this comparison are shown in the Supporting Information.

### 3.1 Voltage Pulsed Atom Probe Experiments

**FIGURE 6:**
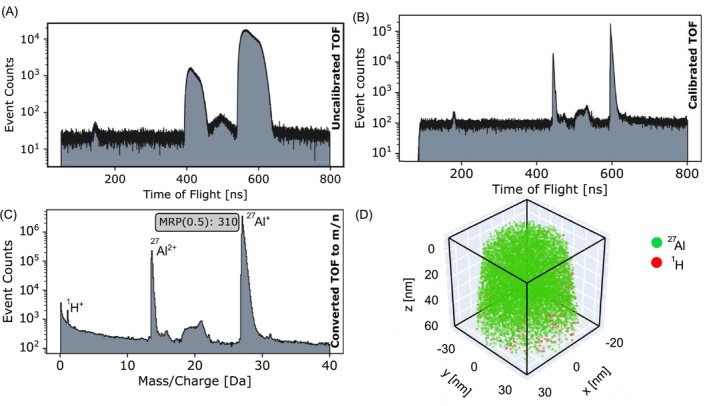
Data calibration and 3D reconstruction. (A) Raw TOF spectrum for Al specimen after ROI selection. (B) Calibrated TOF spectrum using iterative voltage and bowl corrections. (C) Mass spectrum generated from calibrated TOF using parametric conversion. (D) Final 3D reconstruction.

For the Al sample, we collected more than 10 M ions setting the pulse frequency at 200 kHz, pulse fraction of 20%, and detection rate of 0.5%. We also used the Al data to determine the *t*_0_ of the VP atom probe. Figure 6A illustrates the raw TOF spectrum after ROI selection. The raw TOF was then calibrated and converted into a mass spectrum (Figure 6B,C). The obtained MRP(0.5) for Al^+^ is 310. The 3D reconstruction, shown in Figure 6D, was performed using the method proposed by Gault et al. (2011).

**FIGURE 7:**
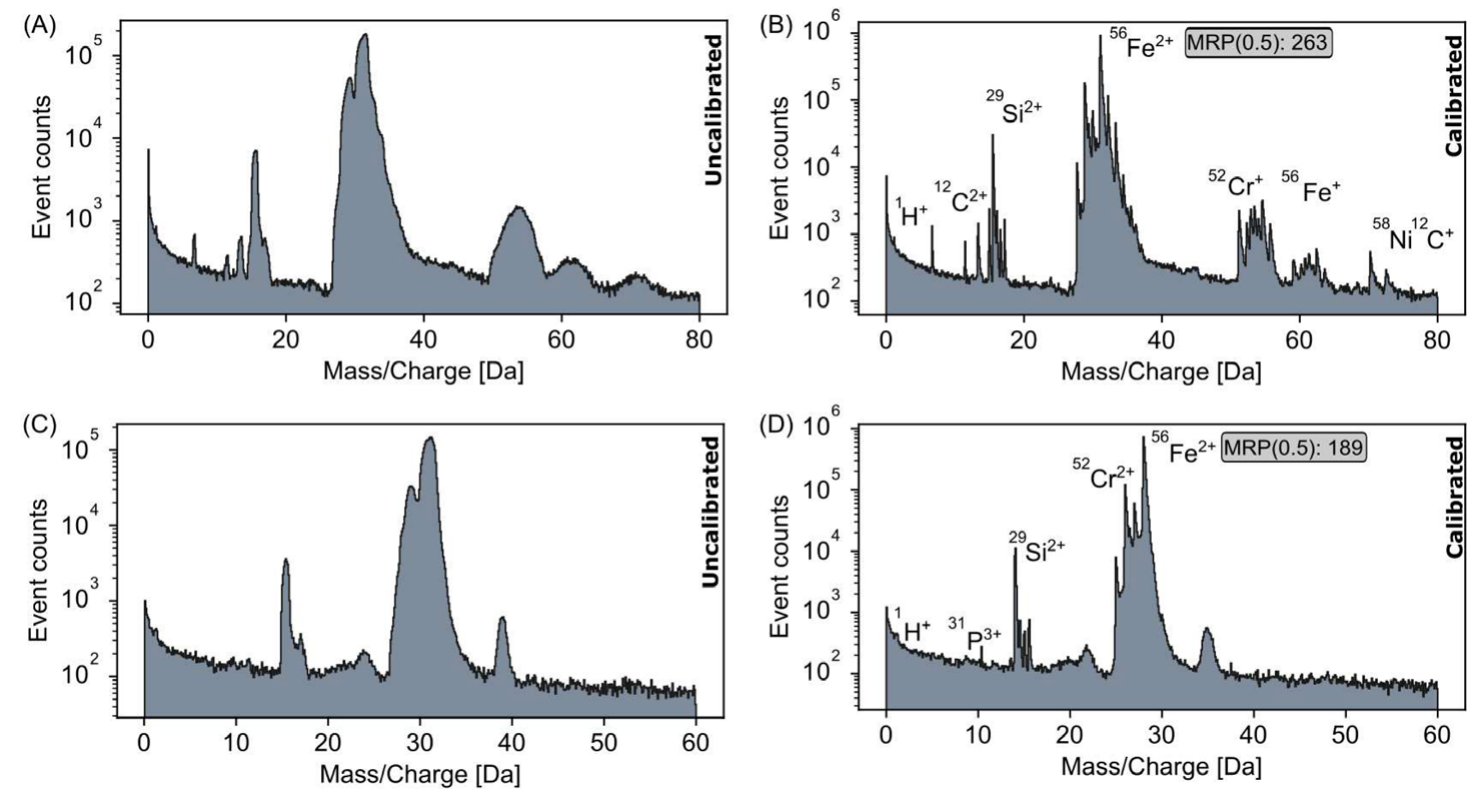
Stainless steels. (A, B) The raw and calibrated mass spectra, respectively, for X14CrMoS17. (C, D) The raw and calibrated mass spectra for X2CrNiMo17‐12‐2.

In the analysis of the stainless steel samples, data were recorded for 4.6 million ions corresponding to X14CrMoS17 and 2.7 million ions from X2CrNiMo17‐12‐2, using the same pulse frequency and detection rates as for the Al sample. The MRP(0.5) values achieved for these datasets were 263 and 189, respectively, as shown in Figure 7. The presence of the titanium analysis chamber resulted in a barely detectable H peak in all three datasets.

### 3.2 Laser‐Pulsed Atom Probe Experiments

**FIGURE 8:**
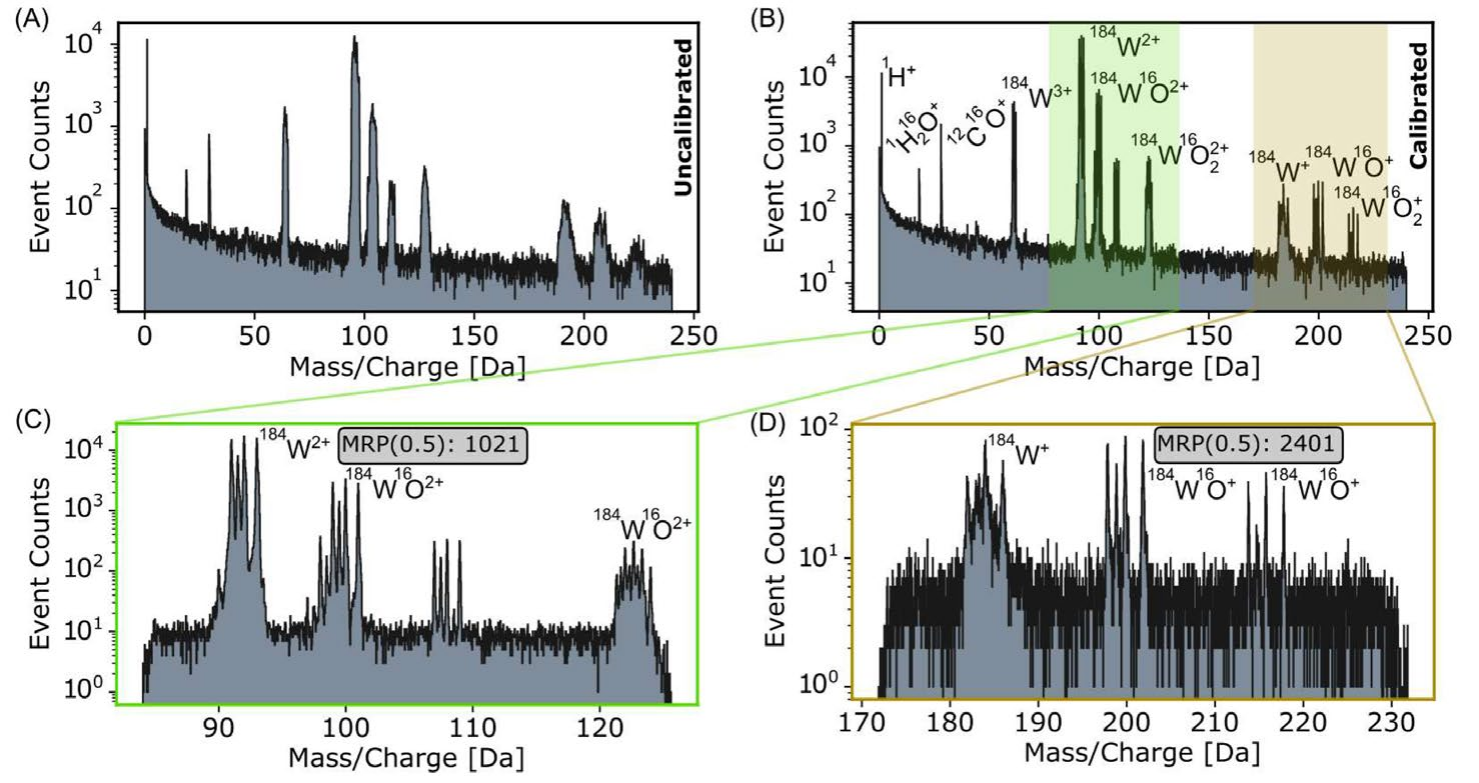
Tungsten measurement; (A) the mass spectrum before calibration for W sample in laser mode. (B) Calibrated mc. (C, D) The green and yellow boxes show a zoom‐in of the W^2+^ and W^+^ isotopes. The MRP(0.5)s are calculated for the main peak ^184^W^2+^ and ^184^W^16^O^+^.

Due to the current limitation of the cooling system in the LP atom probe (refer to Section 2.1), our measurements were limited to room temperature. As a result, we used pure W, with its high melting temperature, that is, the homologous temperature is still low at room temperature. The use of short pulses and an infrared laser resulted in an MRP(0.5) value of 1021 for ^184^W^2+^ and 2401 for ^184^W^+^ (see Figure 8). The pulse frequency was 100 kHz and the detection rate of 1%.

### 3.3 Information Extraction From Detector Raw Data

**FIGURE 9:**
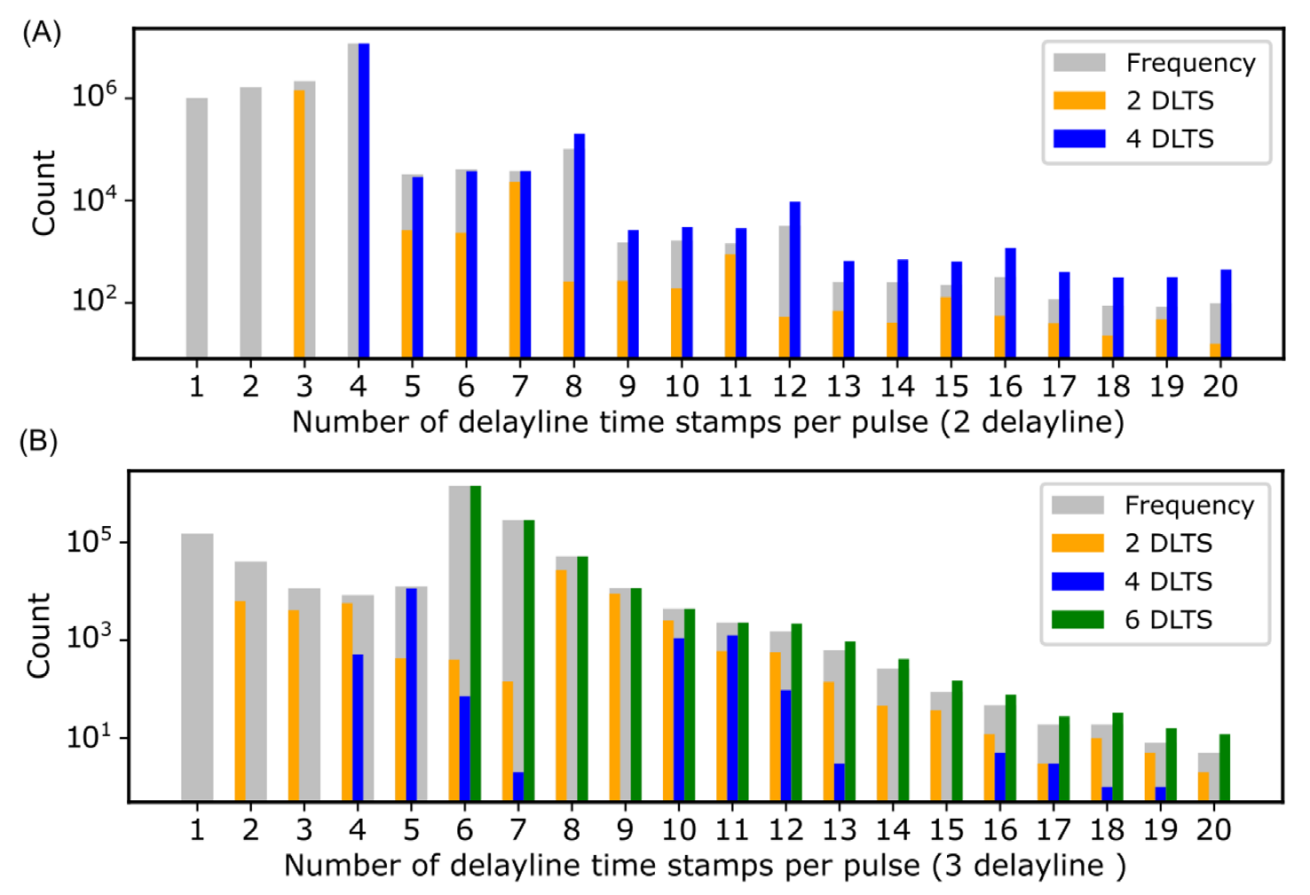
Raw data analysis of two‐delay line and three‐delay line detectors; (A) histogram of the distribution of detected delay line signals (DLTS) per pulse for a two‐delay line detector system (Surface Concept): This histogram illustrates the frequency of recorded DLTS events per pulse for the Al dataset, detailing instances with 1, 2, 3, …, up to 20 DLTS signals per pulse. For each DLTS count, it compares cases where only two DLTS signals were detected to instances where all expected DLTS signals were observed. (B) A corresponding histogram for a three‐delay line detector system (RoentDek), showing the distribution of DLTS per pulse for the W dataset.

In current systems, the delay line end signals, after analog processing in a constant fraction discriminator, are registered by a TDC as delay line timestamps (DLTS), from which TOF (i.e., hit time) and position are calculated. The TOF is the average of the DLTS across all DLTS, while the hit position is proportional to the difference of the DLTS in each delay line. One of the most important possibilities opened up by having full control over the experiment software is direct access to the detector's raw data, that is, the DLTS. With direct access to the data at this level, we can assess detector losses and recover additional information. Here, we present as an example the raw data analysis of Al and W datasets. In a PCB‐based (planar) detector with two‐delay line (*x* and *y*) (Zhu et al. 2016), a uniquely interpretable detector event consists of four timestamps, one on each channel numbered 0–3. A double hit consists of 8, and so on. In Figure 9A, a histogram is shown of the frequency of delay line end times per pulse trigger, excluding triggers with no end times. Although multiples of four are by far the most frequent, pulses with fewer than four DLTS are prominent. While these signals cannot be directly used to calculate position and TOF, they can be used to recover the TOF and one coordinate if two end times for a single delay line are recorded. In addition, they can be used to assess whether lost signals have a physical meaning or are simply detector artifacts.

The analysis of the raw data from the Al dataset showed that 79% of the events consist of all DLTS for both delay lines, *x* and *y*. However, approximately 18.3% of the cases exhibit only one, two, or three DLTS. 2.7% have more than four DLTS (multi hit). Of the events with three DLTSs, 65% give meaningful TOF data, that is, represent hits where one DLTS is missing. Interestingly, for those with only two DLTS, all timestamps represent combinations of both delay lines. If they were caused by random events, half should be registered by either the *x* or the *y* delay line. We therefore have to conclude that most of these are caused by real detector events, where one of the signals per delay line is lost, presumably due to attenuation or the peak detection of the electronics in the delay line. While no interpretation is possible for events with only one timestamp, it must be concluded that a significant proportion (approximately 10% of all events in the Al dataset) are not registered in an interpretable manner by the detection system.

Among events with more than four DLTS, we observed the following: 1.45% of all cases feature multiple hits with both *x* and *y* DLTS. In contrast, 1.25% of the cases involve multiple occurrences of both *x* and *y* DLTS but also include events with missing end times. For instance, while one event might have complete DLTS for all four delay line end pulses, a subsequent event could be missing one DLTS. This absence of data complicates the process of accurately grouping the DLTS, often requiring heuristic decision‐making. One solution is to check all possible four DLTS, calculate the hit position for each group, and exclude results that would lay outside the active detector area. Table 1 shows the percentage of two and four DLTS for each detected ion by both mentioned detectors in Section 2.1.

**TABLE 1:** Comparison of ion detection with missing delay line end times in two and three delay line systems; this table compares the ion percentages in two and three delay line systems (Surface Concept) and two, four, and six delay line systems (RoentDek). The percentages represent the proportion of each ion within the detected real events. In addition, for each ion, the ratio of events where the detector hit position can be calculated to those that are discarded due to missing DLTS is calculated.

Two delay line (Surface Concept)				Three delay line (RoentDek)				
Ion	Two DLTS	Four DLTS	Two/four DLTS	Ion	Two DLTS	Four DLTS	Six DLTS	Two/four + six DLTS
H^+^	0.02%	0.05%	0.400	H^+^	0.02%	0.01%	0.45%	0.043
Al^2+^	14.85%	79.57%	0.186	W^3+^	0.09%	0.03%	8.25%	0.010
Al^+^	0.86%	4.66%	0.184	W^2+^	0.49%	0.33%	90.34%	0.005

By calculating the TOF from single delay line data, we quantified the real events in the raw data missing one delay line in a two‐delay line system or one/two delay lines in a three‐delay line system, showing that approximately 15% of all real events have missing DLTSs. These events must be excluded from the final dataset due to the absence of one of the detector hit coordinates. One advantage of this analysis is the ability to directly estimate the number of missed real events, as the TOF can be calculated based on the information from a single delay line. As a result, we gain a clearer understanding of the proportion of missed events. We also examined the distribution of two DLTS across the *x* and *y* delay lines and found that the *x* delay line has 1.9 times higher occurrence than the *y* delay line. This discrepancy may be due to factors such as variations in the delay line manufacturing process or be rooted in the stacking order of the delay lines. We did a similar analysis for a three‐delay line system that has wire‐wound delay lines (Jagutzki et al. 2002), as shown in Figure 9B. Adding a third delay line increases the likelihood of detecting events, as it allows for decrease in the detector dead time and more precise calculations of the TOF and hit position compared to systems with only two delay lines.

Table 1 summarizes the percentages of events with two and four DLTS for the Al dataset, as well as two, four, and six DLTS for the W dataset, offering insights into the extent of missing delay line signals of each ion and charge state across two distinct detector systems and materials. The ratios of two DLTS to four in two‐delay line systems and two to four and six in three‐delay line systems are also provided in Table 1. Notably, the rate of missing DLTS is higher for hydrogen ions compared to other ions in both systems. This ratio difference needs further investigation, which is beyond the scope of this work.

## 4 Conclusions

In this work, we introduced PyCCAPT, an open‐source Python package designed for atom probe instrument control, data acquisition, and processing. By using modern programming practices and accessible hardware, PyCCAPT offers a highly customizable and transparent alternative to proprietary commercial APT systems. It provides full control and unrestricted access to every stage of the analysis pipeline, a feature we demonstrate to be crucial for addressing detector losses and potential data biases. We also detailed the underlying algorithms of PyCCAPT's data processing, calibration, and electronic delay calculations, showing how they differ from existing methods. Our results demonstrate the effectiveness of PyCCAPT with two commercially available from TDCs Surface Concept and RoentDek, illustrating its versatility across different hardware setups.

When combined with previously available open data analysis tools (Heller et al. 2025; Smith and Young 2023), PyCCAPT forms an open‐source solution for APT. Future developments for PyCCAPT will focus on incorporating laser tracking functionality, improving user documentation and tutorials for greater accessibility, and exploring advanced data analysis methods, addressing the need for experimental control and data calibration. Although it cannot be used for data acquisition with commercial systems, its calibration module can be used for direct‐flight‐path systems (see Supporting Information) and adapted for reflection‐based instruments (Heller et al. 2025). PyCCAPT also enhances transparency in calibration, giving users more control over corrections. Future developments will focus on laser tracking, improved documentation, and advanced data analysis.

## Author Contributions

**Mehrpad Monajem:** conceptualization, methodology, software, data curation, formal analysis, validation, investigation, visualization, project administration, writing – review and editing, writing – original draft, supervision. **Benedict Ott:** methodology, writing – review and editing, validation, investigation. **Jonas Heimerl:** investigation, validation, writing – review and editing. **Stefan Meier:** investigation. **Peter Hommelhoff:** investigation, funding acquisition, project administration. **Peter Felfer:** project administration, funding acquisition, writing – review and editing, methodology, supervision, resources.

## Conflicts of Interest

The authors declare no conflicts of interest.

## Supporting information


**Data S1.** Supporting Information.

## Data Availability

The data that support the findings of this study are available from the corresponding author upon reasonable request.

## Endnotes

^1^

https://pyccapt.readthedocs.io/.
