# Supplementary material for: PyCCAPT: A Python Package for Open‐Source Atom Probe Instrument Control and Data Calibration
Source: Microsc Res Tech. 2025 Jul 19;88(12):3199–210. doi: 10.1002/jemt.70011 (PMC12584301; doi:10.1002/jemt.70011)
Supplement: Supplementary file 1 — Data S1. Supporting Information. [file JEMT-88-3199-s001.pdf]

PyCCAPT: A Python Package for Open-Source Atom Probe Instrument Control and Data Calibration

Mehrpad Monajem, Benedict Ott, Jonas Heimerl, Stefan Meier, Peter Hommelhoff, Peter Felfer

Supporting Information

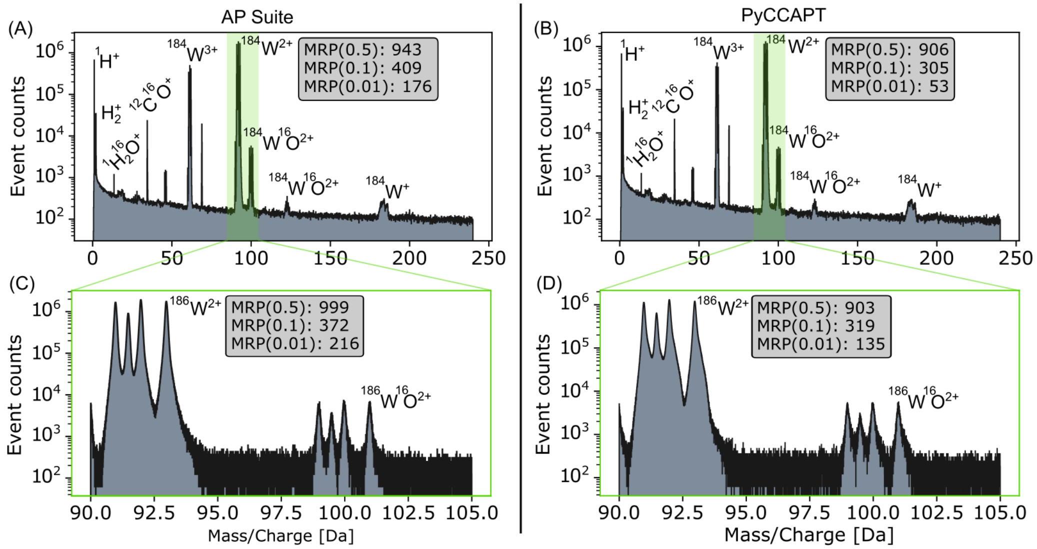

FIGURE 1 Calibration comparison: AP Suite vs. PyCCAPT for W dataset (13.7 M ions): (A) and (B) present the mass spectrum of W dataset obtained using the LEAP 5000 XS, calibrated with AP Suite 6.3.1.110. The same dataset was calibrated using PyCCAPT. MRPs are for $^{184}W^{2+}$ in (A) and (B) and for $^{186}W^{2+}$ in (C) and (D).

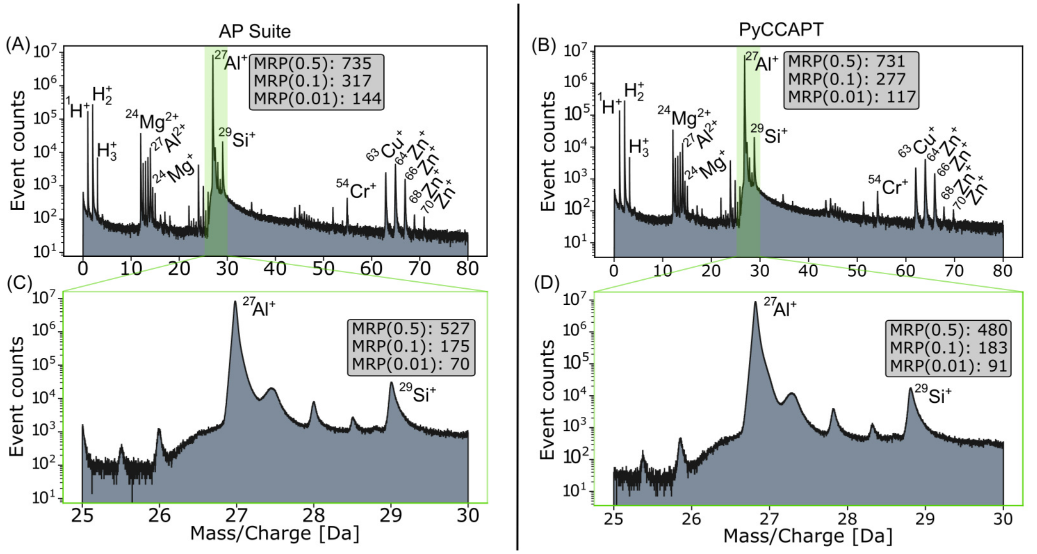

FIGURE 2 Comparison of calibration methods: AP Suite vs. PyCCAPT for a 6xxx series al-Alloy dataset (41.2 M ions): (A) and (B) show the mass spectrum of a 6xxx series Al-alloy, acquired with the LEAP 5000 XS, and calibrated using AP Suite 6.3.1.110. The same dataset was calibrated using PyCCAPT, converting TOF to mc values. MRPs were computed for $^{27}Al^{+}$ in (A) and (B), and for $^{29}Si^{+}$ in (C) and (D).

apt		
**Name**	**Shape**	**Unit and Description**
id	(n,)	N/A, uint64: Experiment loop iteration counter
num_event	(n,)	N/A, uint32: Number of detected events
num_raw_signals	(n,)	N/A, uint32: Number of detected delayline signals
temperature	(n,)	K, float64: Measured temperature of sample
exp_vacuum	(n,)	mBar, float64: Vacuum level in the experiment chamber
timestamps	(n,)	UNIX, float64: time of data recording (microsecond accuracy)
**dld**		
**Name**	**Shape**	**Unit and Description**
x	(n,)	cm, float64: Detector x hit position for the detected event
y	(n,)	cm, float64: Detector y hit position for the detected event
t	(n,)	ns, float64: Time-of-flight for the detected event
high_voltage	(n,)	V, float64: DC voltage value of the power supply
voltage_pulse	(n,)	V, float64: Pulse voltage
laser_pulse	(n,)	pJ, float64: Laser pulse energy
start_counter	(n,)	N/A, float64: Start counter data description
**tdc (raw data) Surface Concept TDC**		
**Name**	**Shape**	**Unit and Description**
start_counter	(n,)	N/A, uint64: Start counter of TDC
channel	(n,)	N/A, uint32: Description of channel data
time_data	(n,)	N/A, uint64: Description of time data
high_voltage	(n,)	V, float64: Applied DC voltage for TDC events
voltage_pulse	(n,)	V, float64: Pulse voltage
laser_pulse	(n,)	pJ, float64: Laser pulse energy
**tdc (raw data) RoentDek TD**		
**Name**	**Shape**	**Unit and Description**
ch0	(n,)	N/A, uint64: Time counter at channel 0 for TDC events, dld 1
ch1	(n,)	N/A, uint64: Time counter at channel 1 for TDC events, dld 1
ch2	(n,)	N/A, uint64: Time counter at channel 2 for TDC events, dld 2
ch3	(n,)	N/A, uint64: Time counter at channel 3 for TDC events, dld 2
ch4	(n,)	N/A, uint64: Time counter at channel 4 for TDC events, dld 3
ch5	(n,)	N/A, uint64: Time counter at channel 5 for TDC events, dld 3
ch6	(n,)	N/A, uint64: Time counter at channel 6 for TDC events, pulse trigger
ch7	(n,)	N/A, uint64: Time counter at channel 7 for TDC events
voltage_pulse	(n,)	V, float64: Pulse voltage
laser_pulse	(n,)	pJ, float64: Laser pulse energy

TABLE 1 HDF5 data structure of the PyCCAPT control module; The dataset file contains four subgroups. The ’apt’ subgroup contains analysis chamber data for each control iteration. The ’dld’ subgroup contains detected events. The remaining two groups store detector raw data, which are explicitly based on the detector type.

Name	Shape	Unit	Description
x (cm)	(n,)	nm, float64	Reconstructed x position in nanometers
y (cm)	(n,)	nm, float64	Reconstructed y position in nanometers
z (nm)	(n,)	nm, float64	Reconstructed z position in nanometers
mc (Da)	(n,)	Da, float64	Calibrated mass-to-charge ratio in Daltons
mc_uc (Da)	(n,)	Da, float64	Uncalibrated mass-to-charge ratio in Daltons
high_voltage (V)	(n,)	V, float64	DC voltage value of the power supply
pulse	(n,)	V or pJ, float64	Pulse voltage or laser power
t (ns)	(n,)	ns, float64	Uncalibrated time-of-flight in nanoseconds
t_c (ns)	(n,)	ns, float64	Calibrated time-of-flight in nanoseconds
x_det (cm)	(n,)	cm, float64	Detector x hit position of ions
y_det (cm)	(n,)	cm, float64	Detector y hit position of ions
delta_p	(n,)	N/A, uint32	Number of pulses since the last detected event
multi	(n,)	N/A, uint32	Detected ions for each pulse
start_counter	(n,)	N/A, float64	TDC counter value

TABLE 2 HDF5 file structure for the PyCCAPT calibration module; This file contains the final results, similar to ATO or EPOS.

Name	Shape	Unit	Description
name	(n,)	N/A, string	Ions name in simple format
ion	(n,)	N/A, string	Ions name in LaTeX format
mass	(n,)	Da, float64	Mass-to-charge ratio based on element's weight and complexity
mc	(n,)	Da, float64	Peak location of mass-to-charge ratio in the dataset
mc_low	(n,)	Da, float64	Lower bound of the mass-to-charge ratio for the peak
mc_up	(n,)	Da, float64	Upper bound of the mass-to-charge ratio for the peak
color	(n,)	N/A, string	Color of the peak in hex format
element	(n,)	N/A, list of string	List of elements in the peak
complex	(n,)	N/A, list of uint32	Complexity of the element (integer)
isotope	(n,)	N/A, list of uint32	Isotope list of the element
charge	(n,)	N/A, uint32	Charge of the element

TABLE 3 HDF5 file structure for PyCCAPT range file.
